# A Case for Participatory Disease Surveillance of the COVID-19 Pandemic in India

**DOI:** 10.2196/18795

**Published:** 2020-04-16

**Authors:** Suneela Garg, Nidhi Bhatnagar, Navya Gangadharan

**Affiliations:** 1 Maulana Azad Medical College Delhi University Delhi India

**Keywords:** participatory, surveillance, COVID-19, pandemic, outbreak, public health, infectious disease, India

## Abstract

The coronavirus disease pandemic requires the deployment of novel surveillance strategies to curtail further spread of the disease in the community. Participatory disease surveillance mechanisms have already been adopted in countries for the current pandemic. India, with scarce resources, good telecom support, and a not-so-robust heath care system, makes a strong case for introducing participatory disease surveillance for the prevention and control of the pandemic. India has just launched Aarogya Setu, which is a first-of-its-kind participatory disease surveillance initiative in India. This will supplement the existing Integrated Disease Surveillance Programme in India by finding missing cases and having faster aggregation, analysis of data, and prompt response measures. This newly created platform empowers communities with the right information and guidance, enabling protection from infection and reducing unnecessary contact with the overburdened health care system. However, caution needs to be exercised to address participation from digitally isolated populations, ensure the reliability of data, and consider ethical concerns such as maintaining individual privacy.

Emerging diseases challenge public health and should be detected early with immediate response taken to control the spread. Disease surveillance forms an essential mechanism for understanding disease epidemiology and provides a sound basis to initiate control measures. Surveillance is the continuous, systematic collection, collation, analysis, and interpretation of health-related data needed for the planning, implementation, and evaluation of public health practices [1].** **Most conventional surveillance systems are not designed to meet challenges posed by pandemics like the coronavirus disease (COVID-19). Fortunately, in the age of technology, mobile phone networks are serving as a major backbone across sectors like finance and banking, transportation, trade, commerce, education, social welfare, public administration, and entertainment. It is time we update our surveillance tool to control the current pandemic and prevent any such occurrences in the future.

Participatory disease surveillance (PDS) is an innovative tool for surveillance of communicable diseases in which citizens are engaged actively for the self-reporting of symptoms or events to help public health experts aggregate and analyze data for appropriate public health intervention [2,3]. These systems are Web 2.0, emphasizing user-generated content, ease of use, participatory culture, and interoperability for end users. They can be supplemented with software like geographical information systems, disease modeling systems, and other analytical software for better analysis of collected data [4]. Digital data through PDS can be used for studying infectious disease dynamics such as early detection of disease outbreaks by continuously monitoring disease trends. Internet-based data from social media can provide researchers with an additional method for examining the period before an outbreak and assess disease-relevant and health-related behaviors [5]

Smart phones have been deployed extensively as disease surveillance tools in public health programs. The Global Observatory of eHealth has defined mobile health (mHealth) as medical or public health practice supported by mobile devices such as mobile phones, patient monitoring devices, PDAs, and other wireless devices [6]. These have been established as effective, affordable, adaptive, and cost-effective tools aiding real time data capture. With the growing telecom sector, which is the second largest in the world, mHealth offers a promising solution for many challenges in the health sector. The Telecom Regulatory Authority of India reports 1183.41 million wireless subscribers with the internet subscriber base being 493.73 million in 2019 [7].

The above statistics make a strong case for the introduction of PDS in situations where our routine surveillance capacities are overwhelmed. Expansion of surveillance capacity is required for COVID-19, as it needs to focus on asymptomatic individuals for the quick detection of symptoms, individuals in quarantine, suspected and symptomatic individuals in isolation, and workers involved in health care delivery at all levels.

A model of PDS has already been established in various countries. Influenzanet was established, covering ten European countries, to monitor influenza-like illness (ILI) and foodborne illness. Similar surveillance systems were used effectively in Australia (Flutracker) and the United States (Flu Near You) to capture data on a weekly basis to determine the trend of ILI. Thailand launched a mobile app DoctorMe in 2014 to track ILI and has 15,000-50,000 registered users reporting symptoms on a daily basis. Mo-Buzz was launched recently in Sri Lanka in 2016 to identify dengue mosquito breeding sites and environmental pollution. It was used to track vector-borne diseases and implement preventive strategies with the help of digital connectivity. The use of such tools helped model and forecast health threats with built-in software and analytics [5,8,9]. These novel technologies and health surveillance data together estimate the range and magnitude of health problems in a community to rapidly detect and respond to outbreaks [10]. The COVID-19 pandemic has burdened health care systems worldwide and emphasized community involvement in monitoring, preventing, and controlling the disease.

Countries have introduced mobile-based apps and digital platforms to aid surveillance activities for COVID-19 control [11-14] (Table 1). In some countries, the participation is voluntary and in others it is the basis for permitting movement in society. Apps for COVID-19 surveillance are made to perform two complementary functions: syndromic surveillance and contact tracing. They have been integrated with sectors beyond health such as law enforcement.

The COVID-19 pandemic in India, with a reproduction number >1 (2-3.5), is still in the second stage of the pandemic, which is feared to progress to the third stage with established community transmission and potential for the disease to spread rapidly in the thickly populated cities and towns of India [15,16]. Currently as of April 7, 2020, there are 4306 active cases and 114 deaths with all states reporting cases. The hot spots are mostly located in densely populated cities and state capitals [17]. The pandemic has been responded to aggressively in India by initiating strong measures of lockdown well in advance. However, disease surveillance needs to strengthen for effective prevention and control of the pandemic in India. The Integrated Disease Surveillance Programme (IDSP) in India is a decentralized surveillance mechanism that uses indicator and event-based surveillance to detect outbreaks early [18]. With the disruption of routine health care services, there is less passive reporting of cases. Active surveillance is being done only for those with travel history and in the form of contact tracing confirmed COVID-19 cases. PDS at the IDSP district hub can support the existing system in locating missed cases.

India has built a coronavirus tracker based on mobile location with the name Aarogya Setu (Figure 1), which translates from Sanskrit to “A bridge of health”. For the first time, the country has introduced a PDS model for any disease. Participation in this platform is voluntary. The core function of the app is risk assessment with the option of reporting oneself to the government. It uses the phone’s location data and Bluetooth to assess the proximity from a person infected with COVID-19 by looking through databases created by the government of lab-confirmed COVID-19 cases. Questions on gender, age, symptom details, comorbidities, travel, and contact history are inquired. The app then scores the risk status of the individual as low, moderate, or high. Individuals are informed on the measures to be taken based on the risk assessment (eg, isolation, log temperature every 2 hours) and given advice for testing with details of control rooms and testing centers available in the individual’s area. It also has a chatbot feature, rolling updates from the health ministry, and helpline numbers for each state in India [19]. In the time of this pandemic, when states have implemented complete lock down, this app is an important mode of communication to address COVID-19-related queries and anxieties. Community awareness on this issue will ensure engagement in the platform; however, the information to download the app and use it must be reinforced by government and health care workers. Major drawbacks at this stage are the optional reporting to the government and an unclear process for contact tracing if a suspected case becomes confirmed. Voluntary participation in the app prevents using it for movement permits and as a basis for taking more strict actions.

**Table 1:** Apps available for coronavirus disease surveillance.

Country	Name of app	Contact tracing	Syndromic reporting	Consent	Geolocation or personal data collected	Comments
China	Alipay Health Code	Yes	Yes	No	Yes	Checklist would issue QR^a^ code with one of three colors denoting quarantine status. The code is checked at various points of movement. Information is shared with the police for appropriate action, if required.
Russia	Social Monitoring	Yes	Yes	No	Yes	Government-issued QR code that needs to be presented to police, if required. It also ensures adequate check on people in quarantine and assesses their compliance with instructions.
South Korea	Corona 100m	Yes	Yes	No	Yes	Demographic data and location history is noted in the app at the time of COVID-19^b^ diagnosis. It also alerts users if they come within 100 m (328 ft) of a location visited by confirmed case.
Singapore	Trace Together	Yes	Yes	No	No	Using Bluetooth, Trace Together identifies other nearby phones with the app during the period of infectiousness for SARS-CoV-2^c^ (14 days). Data is stored in phone for 21 days and accessed only when the person is identified as being in close contact with a confirmed case of COVID-19 or has been diagnosed with COVID-19.
India	Aarogya Setu	No	Yes	Yes	No	Translated in 11 languages for use across all states of India. No mandatory government reporting and functions primarily as an app for self-assessment of COVID-19 risk and information if deemed necessary by an individual.

^a^QR: Quick Response.

^b^COVID-19: coronavirus disease.

^c^SARS-CoV-2: severe acute respiratory syndrome coronavirus 2.

**Figure 1:**
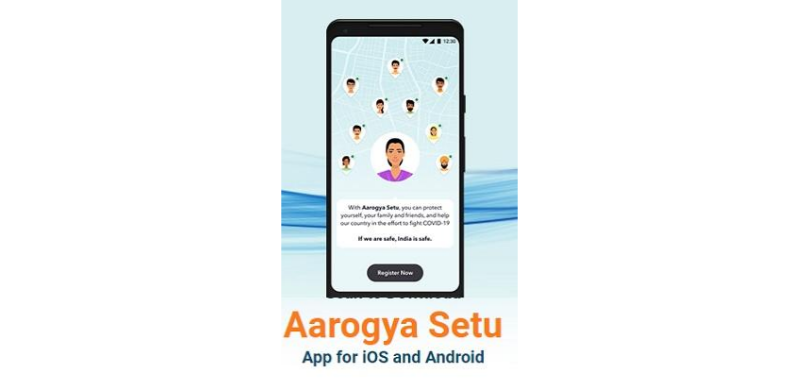
The Aarogya Setu app.

This surveillance system can potentially be used for the prevention and control of the COVID-19 pandemic. It can ensure that information about confirmed and suspected cases in the community is available for the community and government. Geotagging of cases will help in initiating control measures on field situations by the authorities and will inform the community of additional precautions needed. A restrictive testing strategy makes it imperative that data of syndromic reporting is available for the identification and containment of clusters. It will also ensure quality surveillance in areas with overburdened health care systems and resource constraints. It will reflect the trend of ILI and subsequently the disease in the community. The time lag in communication of test results by health authorities can be reduced. The entire process will bring community empowerment with no direct physical contact, adhering to the social distancing regulations currently applicable to the COVID-19 outbreak. These apps or digital platforms can be potentially used in the future to help trained volunteers deliver doorstep diagnostic curative services and implement preventive strategies appropriately. This will prevent patients coming to health care facilities and infecting other people in the process.

The tool of PDS has certain limitations. First, it works on the assumption that volunteers contributing data will be large and representative of the population. Efforts to ensure installation of the Aarogya Setu app must be made actively by the authorities in urban areas for effective surveillance. Initial focus should be given to areas identified as hot spots for transmission, which, until now in India, are mostly in urban areas. Omission of some age groups like older adults and children who use less internet can be overcome by reporting from other household members. However, it should be kept in mind that there will be concerns about populations in rural and remote areas, internet connectivity, availability of smart phones, and digital illiteracy. Second, the main ethical dilemma in cases of PDS is how to ensure adequate protection of participants’ data and ensure proper ethics while obtaining the full benefits of public health surveillance involving digital representative communities of citizens [20]. Third, we also must ensure the ethical use of collected data. Digital surveillance of COVID-19 involves access to personal data and may interfere with individual privacy. It is therefore essential to ensure that the data collected is only used for the purpose of prevention and control of the pandemic, and measures are taken to ensure data security [21].

Thus, the authors strongly promote the use of PDS to support the existing IDSP in India. The tool should be used holistically to assess the behavior of communities toward the pandemic, spread awareness messages, do risk profiling and contact tracing, understand the trend of the disease, and have community-based interventions. One single platform for the state or country will ensure uniformity and participation. Adequate integration with the concerned ministries and organizations involved in the pandemic response should be ensured. The lessons learnt during the PDS of COVID-19 will be useful in future pandemics and will further aid the establishment of routine ILI surveillance in the country.

## Abbreviations

COVID-19: coronavirus disease
IDSP: Integrated Disease Surveillance Programme
ILI: influenza-like illness
mHealth: mobile health
PDS: participatory disease surveillance
